# Colorectal Adenocarcinoma Metastasis to the Tongue

**DOI:** 10.1155/2015/242135

**Published:** 2015-12-02

**Authors:** Kurren S. Gill, Mark A. Frattali

**Affiliations:** ^1^The Commonwealth Medical College, 525 Pine Street, Scranton, PA 18509, USA; ^2^Delta Medix Ear, Nose, and Throat, PC, 940 Jefferson Avenue, Scranton, PA 18510, USA

## Abstract

This case presentation examines a rare clinical entity: colorectal adenocarcinoma (CRC) metastasis to the tongue. CRC is among the least common tumors to metastasize to the oral cavity. Objectives for this case report are to (1) maintain a high index of suspicion for oral cavity tumors representing metastatic disease, (2) consider appropriate surgical and adjunctive interventions, and (3) recognize the significance of identifying the primary tumor via immunohistochemical staining. We present a case of a 57-year-old male with a history of stage IV rectal adenocarcinoma metastatic to the lung who presented to our clinic with a painful mass of the right lateral tongue that he noticed one month before. MRI of the neck revealed a mass involving the anterior two-thirds of the right tongue with irregular margins and an ipsilateral enlarged right jugulodigastric lymph node. The patient underwent right partial glossectomy with primary reconstruction and right modified radical neck dissection. Pathology confirmed poorly differentiated adenocarcinoma consistent with a colorectal primary with lymphovascular and perineural invasion. The tumor was staged as T2N1, and the patient was referred for chemoradiation. In this report, we discuss the presentation, diagnosis, and treatment of this uncommon disease, with a thorough review of the world literature.

## 1. Introduction

Metastasis of primary tumors located elsewhere in the body to the oral and maxillofacial region is rare, representing only 1% of all oral cancers [1]. Oral cavity metastasis typically involves the mandible, with only 16% affecting the oral soft tissues [2]. In the oral cavity, the gingiva is the most common soft tissue site affected, with the tongue being second. Oral metastasis is most commonly from lung, kidney, liver, and prostate in men and from breast, female genital organs, and kidney in women [3]. Rarely does CRC metastasize to the oral cavity, and even rarer to the oral soft tissues. Diagnosis of an oral metastatic lesion typically confers a dismal prognosis, despite treatment with surgery or chemoradiation therapy, and has a median survival of 6 to 8 months [1, 3–5]. When approached with a tumor of the oral cavity, one must maintain a high index of suspicion for a possible metastatic source. In this report, we describe a case of CRC metastatic to the tongue, present the clinical and radiologic features, and discuss treatment options, with a review of the world literature.

## 2. Case Report

A 57-year-old male with a history of stage IV rectal adenocarcinoma metastatic to the lung presented to our outpatient clinic with a painful mass on the right lateral tongue that he noticed one month before.

Elective colonoscopy 2 years before revealed a 2 cm ulcerated polyp at the first rectal fold positive for invasive poorly differentiated primary tumor. Three weeks after his colonoscopy, the patient underwent transanal abdominal robotic total mesorectal excision (TME) with proctosigmoidoscopy. Lymphovascular and perineural invasion were identified. There was no evidence of metastasis at this time and the patient received radiation and chemotherapy.

CT of the chest/abdomen/pelvis eight months after the TME revealed a 1.5 × 0.8 cm spiculated mass in the left lower lung. Pathology confirmed moderately differentiated adenocarcinoma consistent from a colorectal primary tumor. He subsequently underwent video-assisted thoracoscopic surgery (VATS) with left lobectomy. Chemotherapy was offered but it was deferred by the patient.

The patient presented to our clinic 7 months later with a painful mass of the right lateral tongue. Review of systems was negative and the patient denied any weight loss or fatigue. The patient is a nonsmoker.

**Figure 1:**
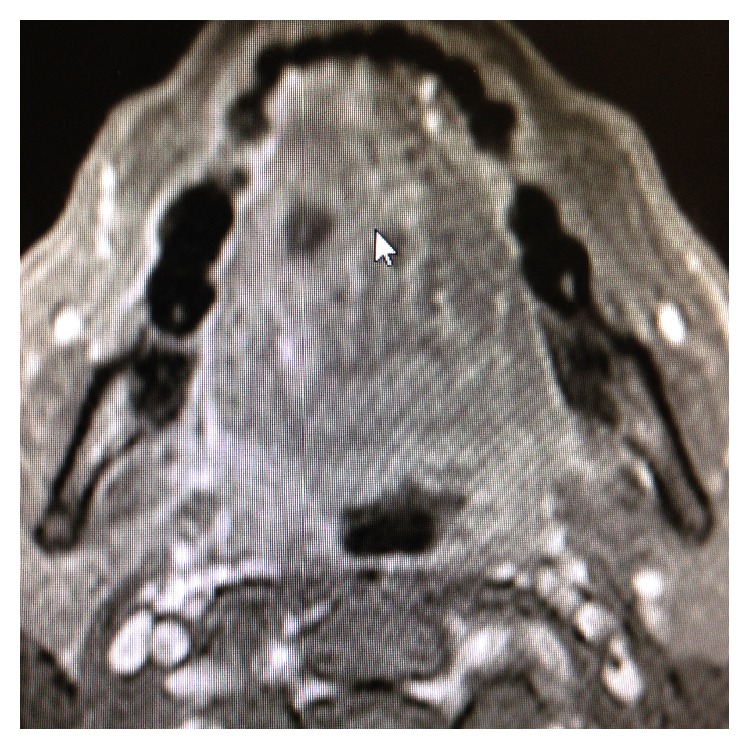
MRI of the neck revealing an anterior tongue mass.

**Figure 2:**
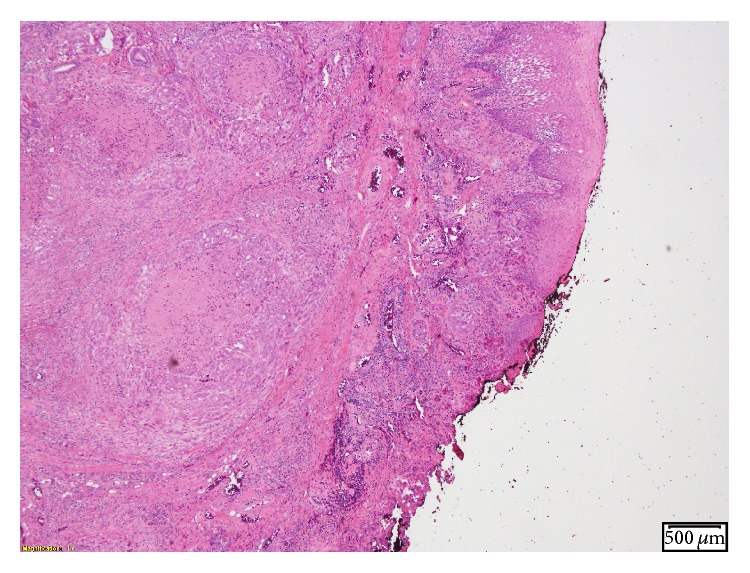
H&E stain showing poorly differentiated adenocarcinoma.

**Figure 3 fig3:**
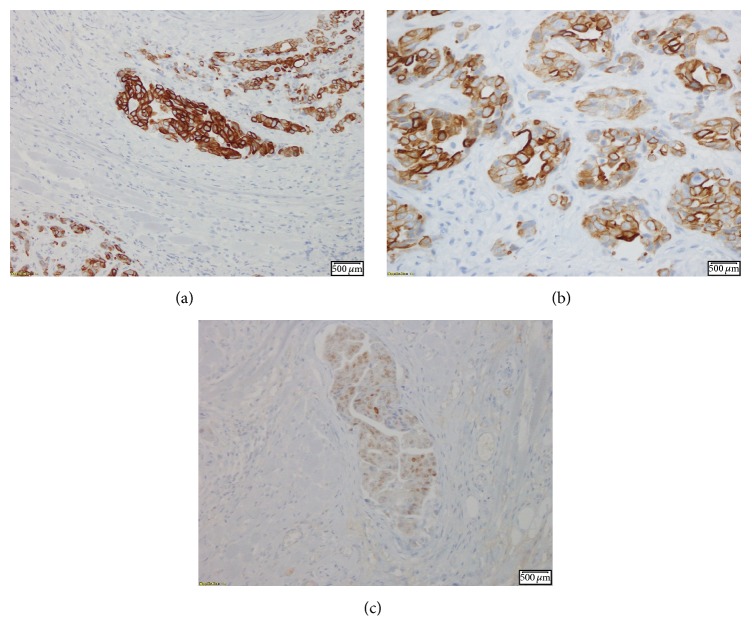
IHC stain showing cytokeratin 20 (a and b) and CDx-2 (c) positivity, markers normally associated with gastrointestinal malignancies.

**Figure 4 fig4:**
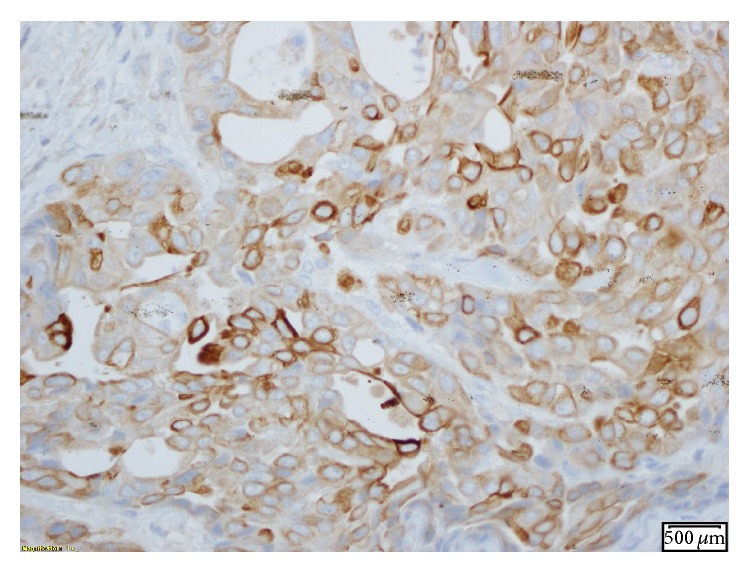
IHC stain positive for CK 5/6.

**Figure 5 fig5:**
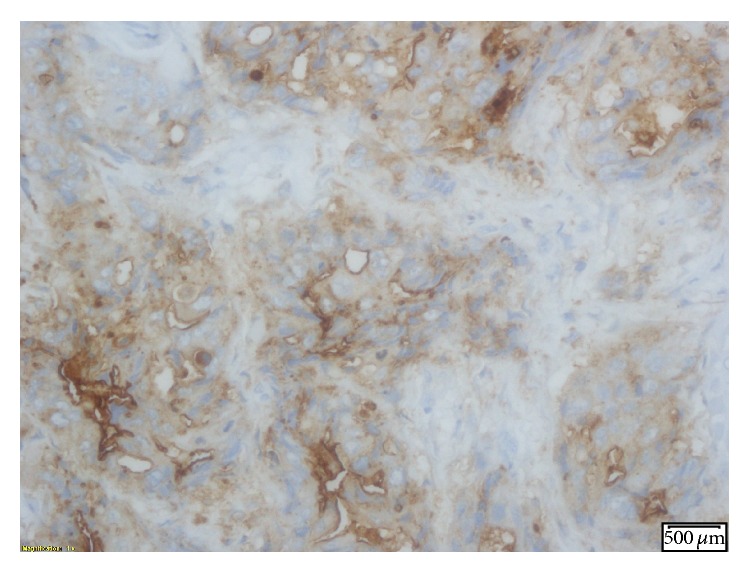
IHC stain positive for polyclonal CEA.

**Figure 6:**
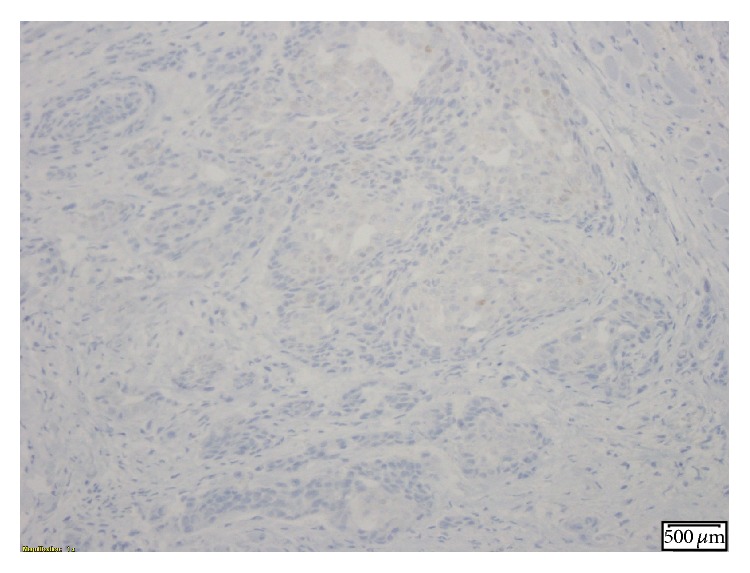
IHC stain positive for P63.

Oral examination revealed fullness of the right tongue; floor of the mouth and buccal mucosa were normal to palpation. MRI of the neck revealed a mass involving the anterior two-thirds of the right tongue with irregular margins and ipsilateral right jugulodigastric lymphadenopathy (Figure 1). Punch biopsies of the tongue mass revealed poorly differentiated adenocarcinoma positive for cytokeratin 20, cytokeratin 5/6, P16, P63, CDx-2, Villin, and polyclonal CEA (Figures 2 [Fig fig3] [Fig fig4] [Fig fig5]–6).

**Figure 7:**
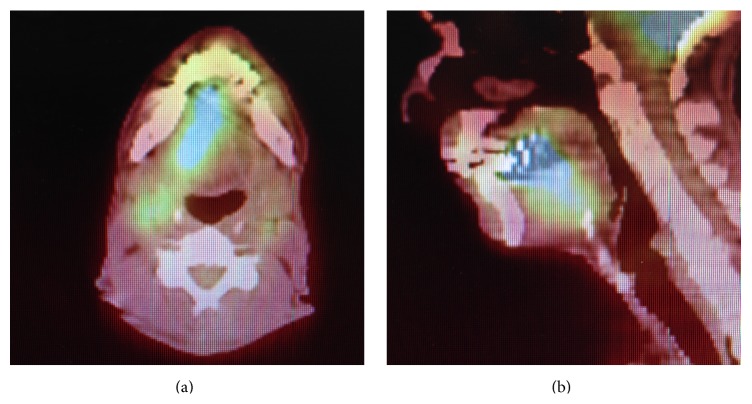
PET scan showing hypermetabolic activity in the tongue and neck.

IHC staining suggested a colorectal primary. Direct laryngoscopy and esophagoscopy revealed a 3 cm mass along the anterior two-thirds of the right tongue which did not cross the midline. It extended to the undersurface of the tongue but did not occupy the floor of the mouth. The posterior tongue was unremarkable. PET scan revealed hypermetabolic activity in the tongue and neck consistent with metastatic disease (Figures 7(a) and 7(b)).

The patient underwent a right partial glossectomy with primary reconstruction and right modified radical neck dissection. Pathology revealed a poorly differentiated adenocarcinoma consistent with a colorectal primary with lymphovascular and perineural invasion. The tumor measured as 3 cm and was staged as T2N1. The patient was referred back to the medical and radiation oncologist for adjunctive treatment. Unfortunately, the patient was unable to complete his chemoradiation therapy and passed away with disease despite all efforts.

## 3. Discussion

Metastasis of primary tumors located elsewhere in the body to the oral and maxillofacial region is rare, representing only 1% of all oral cancers [1]. Analysis of cases of metastatic tumors to the oral region is challenging since most of the information lies in case reports or case series; however, several trends can be extrapolated using the information available.

Oral cavity metastasis classically involves lesions of the mandible, with one study identifying bony involvement in 18/24 patients and soft tissue invasion in only 6/24 patients [1]. Summerlin et al. also found soft tissue metastasis to be present in only 16% of 124 patients [2]. Of the oral soft tissue tumors, the gingiva was the most commonly affected site (54%), followed by the tongue (23%) [3]. A review by Hirshberg and Buchner found that the mobile tongue had a slightly higher incidence of metastasis than the posterior border or tongue base. Increased vascularity to the tongue may lead to its potential to attract metastatic cancer cells [4]. Oral soft tissue metastasis appears to predominate in men versus women (2 : 1) [5] with most patients affected in their fifth to seventh decade [3]. Among the primaries that metastasize to oral soft tissues, only 5% originate from the colon [6]. An analysis of 673 cases of metastatic tumors to the oral cavity by Hirshberg et al. found that the most common primary tumors that metastasize to the oral soft tissues are kidney (14%) in men and genital organs (15%) in females [3]. Recurrence of CRC is typically expected in the liver, peritoneum, pelvis, and lung [7], instead of the oral cavity.

Several theories attempt to explain the mechanism of metastasis to the oral cavity. Batson postulated that the valveless vertebral venous plexus serves as a conduit for bypassing filtration through the lungs and the increase in intrathoracic pressure directs blood flow towards the head and neck region from the caval and azygos venous systems [8]. In a case report describing palatine tonsillar metastasis of rectal adenocarcinoma, Wang and Chen proposed a combination of hematogenous dissemination and retrograde cervical lymphatic spread through the thoracic duct to be responsible for metastasis to the oral cavity [9].

Oral soft tissue metastasis typically manifests as highly vascularized and hemorrhagic exophytic lesions and may be confused for pyogenic granuloma, peripheral giant cell granuloma, or fibrous epulis [5]. Differentiating benign from malignant lesions ultimately relies on histopathologic study. However, various clinical signs, if present, suggest malignancy such as a rapidly evolving lesion, bleeding tendency, an ulcerated or necrotic appearance, mechanical alterations caused by tumor progression, and an overall declining health status of the patient [5]. Furthermore, oral metastatic lesions can cause progressive discomfort, pain, superinfection, dysphagia, interference with mastication, and disfigurement [3]. Metastatic lesions in the oral soft tissues are more easily recognized than mandibular lesions where metastatic deposits may not be evident [3], aiding in early diagnosis and intervention of soft tissue tumor metastasis. The differential diagnosis of a nonhealing, indurated ulcer in the oral cavity includes trauma, malignancies such as lymphoma and squamous cell carcinoma, primary syphilis, and drug-induced lesions caused by the antianginal Nicorandil [10].

Immunohistochemical staining, in conjunction with histopathology, helps aid in definitively differentiating a primary intraoral malignancy from a metastatic lesion with a distant primary tumor. Positive staining for cytokeratin 20 (CK20) suggests a gastrointestinal primary tumor, as most CRCs are positive for CK20 [9]. CK20 and the large bowel marker CDx-2 were both positive in our patient, suggesting the primary source of the tongue adenocarcinoma to be colorectal in origin.

Most patients presenting with oral metastasis have a distant primary tumor already diagnosed and treated. However, van der Waal et al. found that, in 8/24 of patients, the oral lesion was diagnosed before the primary tumor, and in 3 of these patients, identification of the primary tumor remained unknown despite further investigations [1]. They also found that most patients who present with oral metastasis have already developed systemic spread at other sites. The average time between diagnosis of the primary tumor and detection of oral metastatic disease was about 40 months [3]. These findings confer an inherently poor prognosis, leaving palliative care as the only management option in many cases.

Local treatment of mandibular metastasis is nearly always by radiotherapy, whereas soft tissue metastasis is usually approached surgically [1]. A literature review by Zachariades found that the most popular treatment for oral metastasis was radiation followed by no treatment at all, chemotherapy, surgery, or a combination of radiation and chemotherapy [11]. For widespread metastatic disease, Keller and Gunderson suggest radiation therapy as a palliative measure, with the dose and duration of radiation depending on the patient's life expectancy [12]. Despite treatment, the prognosis remains dismal, with median survival time of 6 to 8 months [1, 3, 5]. In several cases in which the oral tumor was the only metastatic lesion, resection appeared to improve the disease-free interval [3], but treatment benefits overall were still palliative at best [5].

Due to the relatively uncommon occurrence of intraoral malignant soft tissue tumors, clinicians should maintain an index of suspicion for a metastatic origin. Further research is needed to elucidate mechanisms of distant tumor metastasis to the oral cavity, which may in turn inspire new treatment approaches and potentially improve survival outcomes.

## References

[1] van der Waal R. I. F., Buter J., van der Waal I. (2003). Oral metastases: report of 24 cases. *British Journal of Oral and Maxillofacial Surgery*.

[2] Summerlin D. J., Tomich C. E., Abdelsayed R. Metastatic disease to the jaws.

[3] Hirshberg A., Shnaiderman-Shapiro A., Kaplan I., Berger R. (2008). Metastatic tumours to the oral cavity—pathogenesis and analysis of 673 cases. *Oral Oncology*.

[4] Hirshberg A., Buchner A. (1995). Metastatic tumours to the oral region: an overview. *European Journal of Cancer Part B: Oral Oncology*.

[5] Murillo J., Bagan J. V., Hens E., Diaz J. M., Leopoldo M. (2013). Tumors metastasizing to the oral cavity: a study of 16 cases. *Journal of Oral and Maxillofacial Surgery*.

[6] Solomon M. P., Gormley M., Jarrett W., Rosen Y. (1975). Metastatic lesions to the oral soft tissues. *Journal of Oral Surgery*.

[7] Spinelli G. P., Caprio G., Tomao F. (2006). Metastatic infiltration of adenocarcinoma of the rectum in hard palate: report of a case and a review of the literature. *Oral Oncology Extra*.

[8] Batson O. V. (1990). The function of the vertebral veins and their role in the spread of metastases in prostatic carcinoma. *BJU International*.

[9] Wang H., Chen P. (2013). Palatine tonsillar metastasis of rectal adenocarcinoma: a case report and literature review. *World Journal of Surgical Oncology*.

[10] Bhaskaran A., Harding S., Courtney D. (2011). An unusual presentation of metastatic colon adenocarcinoma in the oral cavity. *Case Reports in Dentistry*.

[11] Zachariades N. (1989). Neoplasms metastatic to the mouth, jaws and surrounding tissues. *Journal of Cranio-Maxillofacial Surgery*.

[12] Keller E. E., Gunderson L. L. (1987). Bone disease metastatic to the jaws. *Journal of the American Dental Association*.

